# Effect of Gut Microbial Enterotypes on the Association between Habitual Dietary Fiber Intake and Insulin Resistance Markers in Mexican Children and Adults

**DOI:** 10.3390/nu13113892

**Published:** 2021-10-29

**Authors:** Jennifer N. Martinez-Medina, Regina Flores-Lopez, Blanca E. López-Contreras, Hugo Villamil-Ramirez, Daniela Guzman-Muñoz, Luis R. Macias-Kauffer, Paola León-Mimila, Omar Granados-Portillo, Blanca E. Del-Rio-Navarro, Francisco J. Gómez-Perez, Carlos A. Aguilar-Salinas, Nimbe Torres, Armando R. Tovar, Samuel Canizales-Quinteros, Sofia Moran-Ramos

**Affiliations:** 1 Unidad de Genόmica de Poblaciones Aplicada a la Salud, Facultad de Química, UNAM/Instituto Nacional de Medicina Genόmica (INMEGEN), Mexico City 14609, Mexico; nazat_martinez@outlook.com (J.N.M.-M.); regina.floreslp@gmail.com (R.F.-L.); blopez@inmegen.gob.mx (B.E.L.-C.); hugo_villamil@hotmail.com (H.V.-R.); daniela.guz.mu@gmail.com (D.G.-M.); luisrmacias@gmail.com (L.R.M.-K.); paov_lemi@yahoo.com.mx (P.L.-M.); cani@unam.mx (S.C.-Q.); 2 Departamento de Fisiología de la Nutrición, Instituto Nacional de Ciencias Médicas y Nutrición Salvador Zubirán, Mexico City 14080, Mexico; ograpo@yahoo.com (O.G.-P.); nimbester@gmail.com (N.T.); tovar.ar@gmail.com (A.R.T.); 3 Hospital Infantil México Federico Gόmez, Mexico City 06720, Mexico; blancadelrionavarro@gmail.com; 4 Departamento de Endocrinología y Metabolismo de Lípidos, Instituto Nacional de Ciencias Médicas y Nutrición Salvador Zubirán, Mexico City 14080, Mexico; francisco.gomezp@incmnsz.mx; 5 Unidad de Investigación en Enfermedades Metabólicas y Departamento de Endocrinología y Metabolismo, Instituto Nacional de Ciencias Médicas y Nutrición Salvador Zubirán, Mexico City 14080, Mexico; carlos.aguilars@incmnsz.mx; 6 Tecnológico de Monterrey, Escuela de Medicina y Ciencias de la Salud, Monterrey 64710, Nuevo León, Mexico; 7 Consejo Nacional de Ciencia y Tecnología (CONACYT), Mexico City 03940, Mexico

**Keywords:** dietary fiber, hemicellulose, insoluble fiber, gut microbiota, enterotypes, school-age children, adults, HOMA-IR

## Abstract

Dietary fiber (DF) is a major substrate for the gut microbiota that contributes to metabolic health. Recent studies have shown that diet–metabolic phenotype effect might be related to individual gut microbial profiles or enterotypes. Thus, the aim of this study was to examine whether microbial enterotypes modify the association between DF intake and metabolic traits. This cross-sectional study included 204 children (6–12 years old) and 75 adults (18–60 years old). Habitual DF intake was estimated with a Food Frequency Questionnaire and biochemical, clinical and anthropometric data were obtained. Gut microbiota was assessed through 16S sequencing and participants were stratified by enterotypes. Correlations adjusting for age and sex were performed to test the associations between dietary fiber components intake and metabolic traits. In children and adults from the *Prevotella* enterotype, a nominal negative correlation of hemicellulose intake with insulin and HOMA-IR levels was observed (*p* < 0.05), while in individuals of the other enterotypes, these associations were not observed. Interestingly, the latter effect was not related to the fecal short-chain-fatty acids profile. Our results contribute to understanding the enterotype influence on the diet–phenotype interaction, which ultimate could provide evidence for their use as potential biomarkers for future precision nutrition strategies.

## 1. Introduction

Dietary fiber (DF) has been described as an important component of diet due to its beneficial effects in the host [1]. Currently, a lower DF intake in combination with an increased consumption of fat, sugar and high-energy foods is associated with a higher prevalence of obesity worldwide [2,3], predisposing people to metabolic alterations such as insulin resistance and type 2 diabetes [4]. Therefore, it has been proposed that higher DF consumption might prevent or aid in the treatment of some of these alterations [5].

Results from cross-sectional and interventional studies show that DF intake has an inverse relationship with insulin resistance markers, type 2 diabetes and coronary heart disease risk [6,7]. The mechanisms of viscous and gel-forming fibers, such as the soluble types, include lowering of postprandial glucose and insulin responses, decreasing the absorption rate of certain nutrients as well as modulating gastrointestinal transit time [8]. However, the mechanisms related to the structure of insoluble fibers remain largely under-investigated. Interestingly, although response to fiber has a common metabolic signature within the population, it also varies widely among individuals. This high inter-person variability is likely attributable to biological characteristics, including genetics and other lifestyle factors. Recently, it has been suggested that this heterogeneity also stems from different gut microbial profiles [9].

DF is a major dietary component available for the gut microbiota [9,10]. Thus, its consumption can modify gut microbial composition, their metabolic activities and host metabolism [11,12]. Indeed, accumulating evidence suggests that bacteria or bacterial-derived compounds could be partially responsible for some of the biological effects of DF consumption [9]. Reciprocally, gut microbial profiles can influence the way food components, including DF, are metabolized [13]. Particularly specific colonic bacteria, by possessing different carbohydrate-active enzymes, allow these structures to be degraded and consequently produce a variety of metabolites often not produced by the host [14]. For instance, short-chain fatty acid (SCFA) production, which has been related to the metabolic effects of DF consumption, is influenced by gut microbial profiles [15], reinforcing the notion that diet–phenotype effects might be related to an individual’s gut microbial profile.

Currently, when trying to assess the complexity of the thousands of bacterial species in our gut, one option available is a categorization method that assigns an individual’s microbial profile to certain clusters or groups, called enterotypes. These are named depending on the abundance of signature taxa, such as *Bacteroides* and *Prevotella*, the main identified enterotypes, followed by *Ruminococcaceae* [16,17]. These enterotypes are suggested to be biomarkers of gut ecology and stratification and might allow reduction of the large interindividual microbial variation. Recent studies, mainly in European populations, have found evidence that stratification of individuals according to gut microbial enterotypes or their proxy *Prevotella* to *Bacteroides* ratio allows a better understanding of the relationship between diet and phenotype [18]. Specifically, they showed that fiber-rich diets induce greater weight loss in subjects classified as *Prevotella* enterotype or with a high *Prevotella*-to-*Bacteroides* (P/B), while on a low fiber diet these individuals are more susceptible to weight gain [19,20,21]. However, the effect of enterotypes on the diet–metabolic traits relationship has not been assessed for other ethnic or age groups. Thus, the aim of this study was to assess whether gut microbial enterotypes modify the relationship between DF intake and metabolic traits in Mexican school-age children and adults.

## 2. Methods

### 2.1. Study Sample and Data Collection

Children included in this cross-sectional study are a subsample (*n* = 204) from the “Obesity Research Study for Mexican Children” (ORSMEC), which includes school-age children (6 to 12 years) recruited from a summer camp of Mexican Health Ministry employees (Convivencia Infantil, Sindicato de la Secretaría de Salud) and Hospital Infantil de México Federico Gómez, as detailed elsewhere [22]. For this study, exclusion criteria included self-report of obesity-associated metabolic diseases, such as diabetes, as well as antibiotics use within the past 3 months, while elimination criteria were poorly or unanswered dietary information or +2.5 SD of mean total energy intake.

The adult participants (*n* = 75) were recruited from *Hospital Infantil de México Federico Gómez*. The inclusion criteria consisted of age between 18–60 years and the exclusion criteria included self-report of chronic or obesity-associated metabolic diseases, acute events such as illness or surgery and self-report of antibiotic use within the past three months. The elimination criteria were poorly or unanswered dietary information, implausible energy intake (±2.5 SD of mean total energy intake) or absence of stool sample.

The studies were approved by the Ethics Committee of participant institutions (*Instituto Nacional de Medicina Genómica and Hospital Infantil de México*) and were performed in accordance with the Helsinki Declaration II. An informed consent form was signed by each participating adult. Likewise, parents or guardians of children who assented to participate signed the informed consent form.

All participants were assessed through a self-administered questionnaire. For children, the questionnaire was completed by their parents, while adults completed it by themselves. This questionnaire provided information about socio-demographic factors, family health history, physical activity and dietary habits.

### 2.2. Clinical and Biochemical Measurements

Anthropometric parameters, blood pressure and body composition were measured following standardized procedures as previously described [23]. Centers for Disease Control and Prevention (CDC) 2000 growth charts were used as reference to determine body mass index (BMI) percentiles in children and nutritional status was defined based on CDC criteria, i.e., for children: underweight < 5th BMI percentile, normal weight 5th ˂ 85th BMI percentile, overweight ≥ 85th ˂ 95 BMI percentile and obesity ≥ 95th BMI percentile. Cut-off values for adult population were determined according to WHO criteria: underweight BMI < 18.5 kg/m^2^, normal weight BMI ≥ 18.5 kg/m^2^ to <25 kg/m^2^, overweight BMI ≥ 25 kg/m^2^ to<30 kg/m^2^ and obesity BMI ≥ 30 kg/m^2^.

Blood samples were collected after 8–12 h of fasting. Serum biochemical analysis were carried out as previously described [23]. Lipopolysaccharide (LPS) was quantified by the end-point chromogenic LAL assay according to manufacturer instructions (QCL-1000 kit; Lonza, Walkersville, MD). Insulin resistance was estimated with Homeostatic Model Assessment for Insulin Resistance (HOMA-IR) ((fasting glucose (mg/dL) x fasting insulin (µU/mL))/405).

### 2.3. Assessment of Habitual Dietary Intake

A Food Frequency Questionnaire (FFQ), previously validated in a Mexican population, was completed by individuals or children’s parents to estimate habitual dietary intake over the previous year. The FFQ included 107 food items classified into 27 food groups and with ten frequency options: “never”, “less than 1 time per month”, “1 to 3 times per month”, “1 time per week”, “2 to 4 times per week”, “5 to 6 times per week”, “1 time per day”, ”2 to 3 times per day”, “4 to 5 times per day” and “6 times per day” [24].

Estimations of daily average energy, nutrients and DF components intake were made through the Evaluation System of Nutritional Habits and Nutrient Intake software, based on the food composition tables compiled by *Instituto Nacional de Salud Publica* [25], that include content of total, soluble and insoluble fiber as well as particular structures such as hemicellulose, cellulose and lignin. DF consumption was further standardized to 1000 kcal energy intake, to reduce possible variation and interference in the observed associations [26].

Adequate Intake (AI), established by the Food and Nutrition Board of the Institute of Medicine of the National Academies, was used to assess adequacy of total fiber intake. Cut-off values were as follows: for children of 4 to 8 y ≥ 25 g/d, for 9 to 13 y old boys and girls were ≥31 g/d and ≥26 g/d, respectively. For adults, AI was considered ≥25 g/d for women up to 50 y and ≥21 g/d for those over 50 y, while for men AI was ≥38 g/d up to 50 y and ≥30 g/d for those over 50 y [27].

### 2.4. Gut Microbiota Characterization and Enterotype Stratification

Stool sampling and storage were performed as reported by Lopez-Contreras et al., 2018 [28]. Samples were transferred within 12 h after collection in a cooler with freezer packs and stored at −70 °C when received at the research facility. Stool DNA was extracted using QIAamp DNA Stool Mini Kit (Qiagen, Inc.; Hilden, Germany) for children’s samples, according to manufacturer’s instructions. For adult samples, DNA was extracted with QIAamp Power Fecal DNA kit (Qiagen, Inc.; Hilden, Germany), as the QIAamp DNA Stool Mini kit was no longer available. For all DNA samples the V4 hypervariable region of 16S rRNA gene was amplified using 515F and 806R primers as previously described [28] and sequenced in the MiSeq 2 × 250 platform.

The Quantitative Insights into Microbial Ecology (QIIME v1.9) pipeline was used for sequence processing [29]. First, quality filters were used to remove sequences containing barcode mismatches, ambiguous bases or low-quality reads (Phred quality score < 30). After trimming barcodes, demultiplexing was performed. Operational taxonomic unit (OTU) read counts were calculated using the closed reference OTU picking at 97% identity against the Greengenes database (version 13_08). Potential chimeras were detected with USearch61 and excluded from further analysis. To estimate alpha diversity, rarefactions curves were calculated using QIIME, subsampling in the range of 10–17,000 sequences, with a step of 1000 sequences and ten resamplings on each step. Alpha diversity was evaluated with Observed OTUs as well as with Shannon and Chao1 indexes. Enterotypes were determined according to the method described by Arumugam et al. [16] and available in http://enterotype.embl.de/enterotypes.html (accessed on 19 March 2021). Briefly, after taxonomy assignment, genera represented in the tables were filtered to include only genera whose average abundance across all samples was greater than 0.01%. The resulting genus-relative abundance table was used to calculate the distance matrix with the Jensen–Shannon Divergence metric. This matrix was used as input data for the Partitioning around medoids clustering algorithm with the “cluster” package. The results were assessed for the optimal number of clusters using the Calinski–Harabasz (CH) index. Finally, between-class analysis using the “ade4” package in R was performed to identify the drivers for the enterotypes, as well as to link each sample with its group. Besides the clustering approach, Dirichlet multinomial mixture models (DMMs) analysis was also performed for enterotype analysis [30].

For children (*n* = 204), the enterotype distribution was obtained from an extended dataset analysis of 926 subjects as previously reported [22]. For the adult population (*n* = 75) a separate enterotype analysis was performed considering that gut microbial configuration of school-age children is still immature [31]. In addition, given that the enterotypes algorithm has been reported to be susceptible to small sample size [32], the analysis was carried out over an extended dataset that included individuals’ data from the present study combined with a subsample (*n* = 125) of another Mexican adult cohort described in detail elsewhere [33].

**Figure 1:**
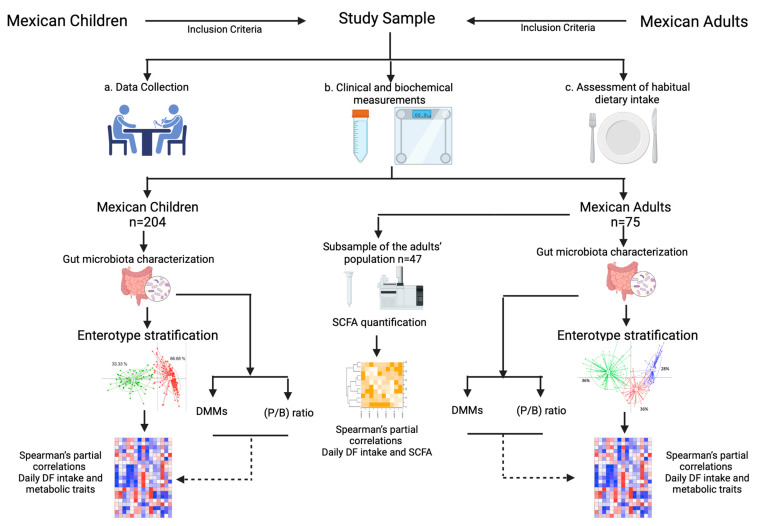
Overview of the workflow for recruitment, data acquisition and analysis. SCFA: Short-chain fatty acids, DMM: Dirichlet multinomial mixture models, P/B: *Prevotella/Bacteroides*. Created with Biorender.com.

Besides enterotypes, a log-transformed *Prevotella* to *Bacteroides* (P/B) ratio was used to stratify participants into high-P/B and low P/B, according to tertiles of the log-transformed P/B ratio [21]. Individuals in the upper tertile were classified as high P/B, while those in the bottom tertile were considered low P/B individuals. This analysis was performed separately for children and adults as shown in Figure 1.

### 2.5. Fecal Short-Chain Fatty Acids Analysis

SCFA were quantified in a subsample of the adult population (*n* = 47) with available fecal samples. Content in fecal samples was analyzed by gas chromatography (Agilent technologies-6850 series 11, Agilent, Santa Clara, CA, USA) with flame ionization detection (Agilent) and using Agilent J & W DB-225 ms column as previously described [34].

### 2.6. Statistical Analysis

Statistical analyses were performed using R and SPSS. Data normality was assessed with the Kolmogorov–Smirnov test. Since data were non-normally distributed, descriptive statistics of the study population were obtained by calculating arithmetic median and interquartile range for continuous variables or percentage for categorical variables. Differences between enterotypes were performed with U Mann–Whitney or with Kruskal–Wallis test with Dunn’s post hoc comparison to ascertain any significant differences between groups. Meanwhile, a chi-squared test was used for categorical data. Partial Spearman correlations were run separately for children and adults, in R through the “ppcor” package. For the correlations between habitual DF intake and anthropometrical variables, age and sex were used as confounding factors. In addition, and given that high body fat mass is associated with metabolic abnormalities [35], correlations of DF components with biochemical variables were further adjusted by body fat percentage. P values were corrected for multiple testing using the Benjamini–Hochberg method as implemented in the p.adjust function in R [36]. Statistical significance was considered when *p* ˂ 0.05.

## 3. Results

### 3.1. Description of Study Sample

The study includes a total of 204 school-age children with a median age of 9.4 y (IQR: 8.38–10.9) and 75 adults with a median age of 39 y (IQR: 34–42). Among children, 6.4% were underweight, 57.4% normal weight and 36.3% were overweight or obese. Meanwhile, among adults 1.3% were underweight, 25.3% normal weight and 73.3% overweight or obese. Median consumption of total DF in children was 18.0 g/d (IQR: 14.1–23.3), while soluble and insoluble fiber median intake was 5.18 g/d (IQR: 3.85–6.92) and 10.3 g/d (IQR: 7.88–13.2), respectively. Total, soluble and insoluble fiber median consumption in adults was 22.8 g/d (IQR: 18.1–28.9), 6.89 g/d (IQR: 5.23–9.69) and 12.7 g/d (IQR: 9.82–15.4), respectively. Adequate intake of total DF was accomplished only by 11.3% of children and 33.3% of adults. Clinical and anthropometric characteristics of the study population, as well as energy intake, are presented in Table 1 and Table 2.

### 3.2. Enterotypes Stratification, Nutrient Intake and Phenotype Differences

We first performed the enterotype analysis following the methodology suggested by Arumugam to stratify the children and adult samples [16]. For children, two enterotypes were previously identified in an extended dataset and were driven by Bacteroides and *Prevotella* abundance [22]. Classification of the current sample participants within these enterotypes showed that 66.7% belonged to Bacteroides while 33.3% belonged to *Prevotella*. Gut microbiota of those within the *Prevotella* enterotype harbored a lower diversity as evaluated by Shannon index when compared to subjects with Bacteroides enterotype (*p* < 0.05).

Anthropometric, clinical and biochemical parameters were not significantly different between children from the two enterotypes, except for triglyceride levels, which were higher in subjects from *Prevotella* enterotype (*p* = 0.001). Moreover, energy and nutrient intake between enterotypes was not significantly different, nor was intake of DF components (Table 1 and Supplementary Table S1).

In contrast to the children’s data, the CH index suggested an optimum of three clusters for the adult sample. These clusters were enriched in *Bacteroides*, *Prevotella* and members of *Ruminococcaceae* family, thus called after the latter taxa (Supplementary Figure S1). Classification of individuals within these enterotypes showed that 36% belonged to Bacteroides, 36% to *Prevotella* and 29% to *Ruminococcaceae*. Consistent with children results, the gut microbiota from individuals classified as *Prevotella* enterotype showed a lower diversity as evaluated by the Shannon index when compared to individuals from the other two enterotypes (*p* < 0.05; Supplementary Figure S2). No significant differences in anthropometric, clinical or biochemical variables were observed among the three enterotypes, except for serum uric acid levels (*p* < 0.05). Energy, nutrient and DF intake were similar among enterotypes (Table 2 and Supplementary Table S2).

### 3.3. Associations between Dietary Fiber Intake and Metabolic Traits in All Participants

We first explored the association of DF intake and its components with metabolic traits, without stratification. In children, consumption of total DF or its components showed no correlation with anthropometric traits. For biochemical variables, only a few significant correlations were observed. Soluble fiber was negatively correlated with HDL cholesterol and weak positive correlations were observed between cellulose intake and serum triglycerides (TG) and between lignin consumption and serum ALT levels (*p* < 0.05) (Supplementary Table S3). In adults, higher intake of insoluble fiber and lignin correlated with lower waist to hip ratio, while hemicellulose consumption showed a moderate correlation with greater body fat (*p* < 0.05). However, none of the previous correlations remained significant after FDR correction. Furthermore, no significant correlations were observed between total DF intake or its components and biochemical variables (Supplementary Table S4).

### 3.4. Associations between Dietary Fiber Intake and Metabolic Traits within Enterotypes

To test whether the enterotypes modify the association between DF intake and the metabolic phenotype, we performed correlations between consumption of the different DF components and metabolic traits within the identified enterotypes in children and adults.

**Figure 2:**
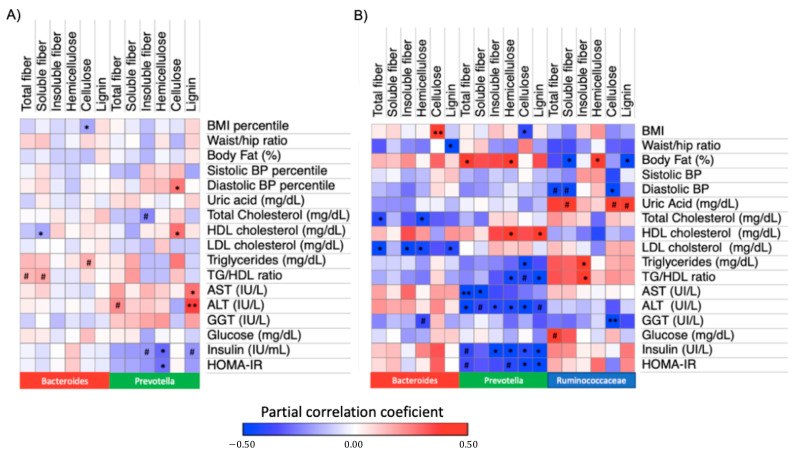
Correlations between DF intake and metabolic traits within enterotypes for (**A**) children and (**B**) adults. Heatmap represents partial Spearman correlations between daily DF intake (adjusted per 1000 kcal) and metabolic traits. Correlations with anthropometric traits were adjusted by sex and age, while correlations with biochemical variables were further adjusted for body fat percentage. # *p* < 0.10, * *p* < 0.05 and ** *p* < 0.01.

In children from *Prevotella* enterotype, no significant correlations were observed with anthropometric traits. However, in these individuals, hemicellulose intake showed negative correlations with serum insulin levels and HOMA-IR, after adjusting for age, sex and body fat. Cellulose intake correlated positively with higher serum HDL cholesterol but also with higher TG levels and diastolic blood pressure percentile. Lignin intake correlated positively with AST and ALT levels (*p* < 0.05; Figure 2A). In contrast, in children from the *Bacteroides* enterotype, only cellulose intake was negatively correlated with BMI percentile and soluble fiber intake was negatively correlated with HDL cholesterol levels (*p* < 0.05; Figure 2A). After FDR correction, only the positive correlation between lignin intake and serum ALT levels in children from the *Prevotella* enterotype remained significant (P_FDR_ < 0.05), while the correlations between hemicellulose intake and insulin resistance markers were close to significance (P_FDR_ < 0.2; Supplementary Table S5).

We then sought to discover whether the associations identified in children were also observed in adults. In *Prevotella* individuals, total DF and hemicellulose intake correlated positively with adiposity (evaluated as body fat percentage), while cellulose intake correlated with negatively with BMI (*p* < 0.05) (Figure 2B). Consistent with the results observed in children, higher intake of hemicellulose correlated negatively with serum insulin levels and as a trend with HOMA-IR (*p* = 0.05) (Figure 2B). Higher intake of this type of DF was also correlated with higher HDL cholesterol and lower serum ALT levels (*p* < 0.05). Intake of total DF, cellulose and lignin showed a negative correlation with serum levels of ALT, insulin and HOMA-IR that were significant or as a trend (*p* < 0.05 or *p* < 0.1, Figure 2B). After FDR correction, the latter correlations remained as a trend (*p* < 0.1) or were close to significance (P_FDR_ < 0.15; Supplementary Table S6).

In adults from Bacteroides enterotype and in contrast to the observed results in children, cellulose intake was positively correlated with BMI (*p* < 0.05), while lignin intake showed a significant negative correlation with waist to hip ratio (*p* < 0.05). Total and insoluble fiber as well as hemicellulose and lignin intake were negatively correlated with LDL cholesterol levels, while higher consumption of total DF and hemicellulose were also negatively correlated with total cholesterol levels (*p* < 0.05, Figure 2B). However, none of the previous correlations remained significant after FDR correction (Supplementary Table S6).

Finally, in individuals within *Ruminococaceae* enterotype, hemicellulose intake was positively correlated with body fat percentage (*p* < 0.05), while soluble fiber and lignin intake correlated negatively (*p* < 0.05). Cellulose intake was negatively correlated with diastolic blood pressure and serum GGT levels, while a positive correlation of insoluble fiber intake with serum TG was observed (*p* < 0.05, Figure 2B). After FDR correction, only the negative correlation between cellulose intake and serum GGT levels remained significant (P_FDR_ < 0.05, Table S6).

### 3.5. Association between Hemicellulose Intake and Insulin Resistance Markets among DMM Enterotypes

Given that gut microbial clustering algorithms for enterotype assignment may yield different results, we performed enterotype analysis based on Dirichlet multinomial mixture models (DMMs), which is suggested to be a statistically more rigorous approach [17]. In children and adults, three enterotypes were identified. The top genus driving community 1 was Bacteroides, for community 2 it was *Prevotella* and for community 3 it was again Bacteroides (Supplementary Figures S3 and S4). Interestingly, when we classified individuals into these enterotypes, most of those initially assigned to *Prevotella* remained in this enterotype (84% of children and 85% of adults). Next, we performed the correlations between dietary fiber intake and metabolic traits. Since most of the correlations did not remain significant after FDR correction with the previous analysis, we focused only on those consistent between children and adults. In adults from *Prevotella* enterotype, hemicellulose was negatively correlated with insulin and HOMA (rho = −0.52, *p* = 0.022 and rho = −0.47, *p* = 0.049, respectively). In children from this enterotype, hemicellulose intake was negatively correlated with insulin (rho = −0.28, *p* = 0.039) and as a trend with HOMA (rho = −0.24, *p* = 0.075). These correlations were not observed in individuals from the other enterotypes.

### 3.6. Association between Hemicellulose Intake and Insulin Resistance Markers Stratifying by Prevotella to Bacteroides Ratio

Given that *Prevotella* and Bacteroides genera have been shown to have the largest variance in terms of relative abundance [17], we sought to discover whether the consistent association between hemicellulose intake and insulin resistance markers was also valid using the *Prevotella* to Bacteroides ratio. The same 68 children initially assigned to *Prevotella* enterotype were in the high P/B group, thus the negative correlations between hemicellulose intake and insulin resistance markers were significant (*p* < 0.05). For adults, 24 out of 27 individuals originally assigned in the *Prevotella* enterotype were classified as high P/B. Within this group, hemicellulose intake showed a negative and significant correlation with insulin (rho = −0.49, *p* = 0.03) and as a trend with HOMA-IR (rho = −0.44, *p* = 0.06), as well as a negative correlation with TG/HDL ratio (rho = −0.45, *p* = 0.05).

### 3.7. Association between Dietary Fiber Consumption and Fecal Short-Chain Fatty Acids (SCFA) among Enterotypes

To test whether differential associations observed between hemicellulose and insulin resistance markers among enterotypes were related to SCFA production, fecal SCFAs were quantified in a subsample of the adult population (*n* = 47). Individuals within the *Prevotella* enterotype showed significant higher fecal levels of propionate, when compared to those in Bacteroides enterotype (*p* = 0.04) (Table 3). In addition, total fecal SCFA levels in *Prevotella* individuals were positively correlated with body fat percentage (rho = 0.45, *p* = 0.03). However, in these individuals, neither hemicellulose intake nor consumption of other types of DF were associated with fecal SCFA levels. In contrast, in participants of the Ruminococcaceae enterotype, soluble fiber intake showed a weak positive correlation with total fecal SCFA (rho = 0.076, *p* = 0.05), while in Bacteroides individuals, higher cellulose consumption correlated with greater fecal butyrate (rho = 0.65, *p* = 0.04). However, these correlations did not remain significant after FDR correction. Among enterotypes, no significant associations were observed between individual or total SCFA and biochemical variables.

## 4. Discussion

The purpose of this study was to assess whether gut microbial profiles evaluated as enterotypes could modify the association between DF intake and the metabolic profile. Interestingly, when we stratified individuals according to the identified enterotypes we consistently observed that in children and adults from *Prevotella* enterotype, hemicellulose intake correlated negatively with markers of insulin resistance, which was not observed in the other enterotypes. Interestingly, the association between hemicellulose and HOMA-IR was not related to fecal SCFA levels.

Observational studies show that high DF consumption has been associated with a decreased risk for different chronic diseases [37]. In our study, when analyzing the whole child sample, we only observed minor associations between consumption of some of the DF components and metabolic traits. This might be related to the dose–response relationship between DF consumption and chronic disease risk [37]. For instance, when considering only the children with an adequate DF intake (*n* = 23), soluble fiber intake was negatively correlated with serum insulin levels and HOMA, however no associations with other dietary fiber components were observed (data not shown). Thus, our results, at least for soluble fiber consumption, are likely influenced by the fact that only 11% of the children fulfill the AI intake for total DF. In adults, although only a third of the adults reached the AI for total DF consumption, the nominal negative correlation between insoluble DF and waist to hip ratio is consistent with other large-scale studies [38].

Enterotypes have been associated with habitual diets with different nutrient intakes [39]. It has been reported that the *Prevotella* enterotype or a major abundance of this genus are associated with carbohydrate-enriched long-term diets in children [40,41,42] and adults [43,44,45]. In contrast, Bacteroides enterotype or its abundance, as well as *Ruminococcaceae* enterotype, have been associated with protein- and fat-enriched diets [44,46,47]. Here, we did not observe significant differences in macronutrient intake between enterotypes in both age groups. Only when using DMM for enterotype assignment, did children classified in community 3, dominated by *Bacteroides*, show higher insoluble DF intake when compared to children from the other two enterotypes. This lack of differences in dietary intake between enterotypes could be related to the nature of our sample, which is composed of individuals from the same geographic region that in general have a lower DF intake and similar dietary patterns. In fact, most previous studies have compared samples from different regions and dietary preferences, where there might also be influence of other environmental factors.

Even though there were no differences in DF intake between enterotypes, we observed differential associations between DF components and metabolic traits among enterotypes. In our study, and despite a modest intake of DF, only in children and adults from *Prevotella* enterotype, did hemicellulose intake show nominal negative associations with insulin serum levels and HOMA-IR, while among children and adults from other enterotypes no consistent associations were observed. Despite the latter correlations not remaining significant after FDR correction, when using DMM models for enterotype assignment, in the *Prevotella*-dominant enterotype the correlations between hemicellulose intake and insulin resistance markers were consistent (Supplementary Table S7). Furthermore, by using the P/B ratio, these associations were also corroborated in children and adults. This is in agreement with a previous report where individuals with high *Prevotella* abundance showed greater improvement in glucose metabolism after a cereal high fiber intervention [48]. Consistently, other studies have also shown that individuals with a high P/B ratio show the greatest metabolic benefit of high fiber interventions while individuals with a low P/B appear to have little or no benefit [18]. Interestingly, those studies also show that in subjects with a high P/B ratio, fiber intake is closely related to HOMA-IR levels [21]. Altogether, it seems that despite the method for enterotype assignment, individuals harboring a *Prevotella* enterotype or a high P/B ratio could be more sensitive to the effects of fiber consumption, particularly for hemicellulose, even at levels below the adequate intake. Although more interventional studies are needed to corroborate the latter results, this could have potential implications to improve efficacy in nutritional interventions.

It has been suggested that differences in taxonomic composition may contribute to functional and ecological differences among enterotypes. An in vitro study showed that the dominance of *Prevotella* in a human fecal inocula produced larger amounts of total SCFA and especially propionate from different fiber structures included in insoluble fibers, compared with fermentation with the *Bacteroides*-enriched sample [49]. Propionate has been described as an intestinal gluconeogenic substrate that activates a gut–brain neural circuit to increase insulin sensitivity and glucose tolerance [50]. Thus, in a subsample of adults, we tested whether the associations between hemicellulose and insulin resistance markers could be related to a differential production of SCFA, the main metabolites produced from dietary fiber degradation. Interestingly, despite higher levels of propionate in adults from the *Prevotella* enterotype, in these individuals, consumption of hemicellulose or any other dietary fiber component was not related to fecal SCFA levels, and the latter were neither associated to insulin resistance markers. This suggests that the association between hemicellulose intake and insulin resistance among *Prevotella* individuals might not necessarily be related to SCFA production. However, there are other mechanisms that might be involved. Indeed, differences among enterotypes include intestinal transit time which likely influences nutrient absorption, bile acid metabolism, as well as production of other bacterial-derived metabolites [17]. For instance, imidazole propionate, a bacterial metabolite produced from histidine and related to higher insulin resistance, showed a negative association with dietary fiber consumption and a differential production pattern among enterotypes [51]. Thus, further studies are warranted to explore whether the mechanisms underlying the observed associations are related to microbial functional differences or if enterotypes only serve as biomarkers of gut physiology.

Finally, it was brought to our attention that in children and adults of *Prevotella* enterotype, associations with metabolic traits were mostly observed for hemicellulose and insoluble fiber components (cellulose and lignin). Even though in general these structures have been described as less viscous and with a minor capacity of gel-forming compared to soluble fibers, properties commonly related to the metabolic effects of DF intake [52,53], our results underscore the need to explore other mechanisms that by interacting with gut microbial profile could contribute to their beneficial effects.

We acknowledge several limitations in our study. First, due to its cross-sectional nature, causality is not demonstrated. Second, the correlations between hemicellulose and insulin resistance markers did not remain significant after FDR correction. Studies that characterize the gut microbiota or *Prevotella/Bacteroides* ratio have described significant correlations between dietary fiber intake and metabolic parameters, particularly in individuals from the *Prevotella* enterotype [19]. Thus, it is plausible that in our study the observed associations are not true false positive results. However, studies with a larger sample size will be necessary to confirm them. Third, quantification of SCFA was performed only in a subsample of adults with available fecal samples which could have influenced the lack of association with dietary fiber consumption. In addition, there were no children’s fecal samples available, thus whether SCFA fecal levels are associated with dietary fiber intake in children warrants further study. Fourth, measurements of other metabolites such as bile acids and imidazole propionate or of gastrointestinal transit time markers might provide additional information to understand the potential mechanisms underlying our findings. In contrast, advantages of our study include the participation of a Latin American population with high prevalence of obesity and metabolic complications where the role of the gut microbiota in the interaction between diet and phenotype remains underexplored. Furthermore, the evaluation of children and adults, with three different models for enterotype assignment or their proxy, strengthen our results and allowed us to replicate the results in different age groups.

In summary, the results presented here suggest that individuals harboring a *Prevotella*-dominant enterotype may have a greater benefit on insulin resistance markers upon consumption of hemicellulose rich foods, even at low intake levels. Despite a certain level of uncertainty, our results contribute to understanding the influence of enterotypes on diet–phenotype interaction, which could ultimately provide evidence for their use as potential biomarkers for future precision nutrition strategies aimed for metabolic complications in children and adults.

## Figures and Tables

**Table 1:** Characteristics of the children included in the study.

	Total Sample (*n* = 204)	Bacteroides (*n* = 136)	Prevotella (*n* = 68)	*p*
Sex; *n* (%)				
Boys	109 (53.4)	69.0 (50.7)	40.0 (58.8)	0.30
Girls	95.0 (46.6)	67.0 (49.3)	28.0 (41.2)	
Nutritional status; n (%)				
Underweight–normal weight	130 (63.7)	91.0 (66.9)	39.0 (57.4)	0.18
Overweight–obesity	74.0 (36.3)	45.0 (33.1)	29.0 (42.7)	
Age (years)	9.40 (8.38–10.9)	9.56 (8.40–10.9)	9.26 (8.30–11.1)	0.98
Waist/hip ratio	0.87 (0.83–0.92)	0.87 (0.83–0.91)	0.89 (0.83–0.93)	0.14
BMI (percentil)	74.7 (31.7–92.4)	68.8 (29.8–90.5)	79.9 (37.0–93.9)	0.19
Body fat (%)	31.5 (24.2–39.1)	30.2 (23.5–38.4)	33.8 (24.8–40.9)	0.20
Sistolic BP (percentil)	45.9 (25.4–69.0)	45.9 (25.0–69.0)	45.9 (27.2–69.0)	0.86
Diastolic BP (percentil)	81.1 (64.4–90.1)	81.5 (66.2–90.3)	80.1 (62.0–89.8)	0.53
Creatinin (mg/dL)	0.46 (0.40–0.51)	0.45 (0.40–0.51)	0.47 (0.40–0.53)	0.13
Uric acid (mg/dL)	4.5 (3.9–5.2)	4.50 (3.90–5.20)	4.50 (4.00–5.50)	0.50
Total cholesterol (mg/dL)	164 (147–184)	163 (144–185)	166 (150–183)	0.41
HDL (mg/dL)	48.0 (42.0–56.0)	47.5 (41.0–56.0)	48.0 (42.0–57.0)	0.99
LDL (mg/dL)	97.0 (83.0–113)	97.5 (84.0–114)	97.5 (82.25–113)	0.75
Triglycerides (mg/dL)	78.0 (54.3–107)	70.5 (51.3–99.5)	91.0 (67.3–134)	**1 × 10^−3^**
AST (IU/L)	29.0 (26.0–34.0)	29.0 (27.0–35.0)	29.0 (26.0–34.0)	0.39
ALT (IU/L)	20.0 (16.0–25.0)	20.0 (16.0–26.0)	19.0 (16.0–24.0)	0.62
GGT (IU/L)	13.0 (11.0–16.0)	13.0 (12.0–16.0)	13.0 (11.0–16.0)	0.45
Glucose (mg/dL)	88.0 (85.0–92.0)	88.0 (85.0–92.0)	89.0 (84.3–93.0)	0.56
Insulin (IU/mL)	5.50 (3.60–7.88)	5.15 (3.45–7.45)	5.85 (3.83–9.03)	0.21
LPS (pg/mL)	0.99 (0.48–1.36)	0.70 (0.51–1.38)	0.82 (0.40–1.23)	0.79
C-reactive protein (mg/dL)	0.07 (0.02–0.17)	0.07 (0.01–0.16)	0.07 (0.02–0.18)	0.61
HOMA-IR	1.17 (0.76–1.77)	1.13 (0.76–1.69)	1.24 (0.82–2.01)	0.23
Total energy intake (kcal/day)	1824.14 (1431.29–2201.73)	1846.04 (1440.87–2154.09)	1809.95 (1406.36–2328.50)	0.85
Total DF (g/1000 kcal/day)	10.4 (8.73–11.8)	10.4 (8.81–11.6)	10.2 (8.47–12.0)	0.86
Soluble fiber (g/1000 kcal/day)	2.95 (2.44–3.53)	3.01 (2.50–3.44)	2.90 (2.37–3.59)	0.55
Insoluble fiber (g/1000 kcal/day)	5.67 (4.94–6.59)	5.69 (4.99–6.59)	5.62 (4.81–6.72)	0.60
Hemicellulose (g/1000 kcal/day)	1.82 (1.53–2.31)	1.82 (1.52–2.29)	1.82 (1.55–2.34)	0.80
Cellulose (g/1000 kcal/day)	2.67 (1.96–4.24)	2.66 (2.00–4.05)	2.89 (1.85–4.45)	0.87
Lignin (g/1000 kcal/day)	0.46 (0.37–0.57)	0.46 (0.38–0.55)	0.45 (0.35–0.60)	0.91
AI of dietary fiber; n (%)	23.0 (11.2)	13.0 (9.56)	10.0 (14.7)	0.27

Values represent median and interquartile range or *n* and percentage. Significant *p*-values are shown in bold. BP: Blood pressure; AST: Aspartate aminotransferase; ALT: Alanine aminotransferase; GGT: Gamma glutamyl transferase; DF: Dietary fiber, AI: Adequate intake.

**Table 2:** Characteristics of adults included in the study.

	Total Sample (*n* = 75)	Bacteroides (*n* = 27)	Prevotella (*n* = 27)	Ruminococcaceae (*n* = 21)	*p*
Sex; n (%)					
M	13.0 (17.3)	5.0 (18.52)	6.00 (22.22)	2.00 (9.52)	0.50
F	62.0 (82.7)	22.0 (81.48)	21.0 (77.78)	19.0 (90.48)	
Nutritional status; n (%)					
Underweight–normal weight	20.0 (26.7)	9.00 (33.3)	5.00 (18.5)	6.00 (28.6)	0.46
Overweight–obesity	55.0 (73.3)	18.0 (66.7)	22.0 (81.5)	15.0 (71.4)	
Age (years)	39.0 (34.0–42.0)	40.0 (35.0–43.0)	38.0 (32.0–41.0)	39.0 (35.0–41.5)	0.30
Waist/hip radio	0.85 (0.81–0.90)	0.85 (0.82–0.90)	0.85 (0.80–0.92)	0.82 (0.79–0.89)	0.54
BMI (kg/m²)	27.8 (24.8–30.4)	26.6 (24.0–29.4)	29.0 (26.3–32.5)	27.1 (24.8–29.5)	0.16
Body fat (%)	35.3 (30.5–41.2)	35.0 (28.6–37.0)	38.5 (33.0–42.5)	35.3 (29.0–43.5)	0.13
Sistolic BP (mmHg)	113 (101–122)	113 (93.5–118)	114 (101–126)	116 (103–122)	0.57
Diastolic BP (mmHg)	73.0 (67.0–79.3)	76.0 (61.0–81.0)	71.0 (67.8–78.0)	73.0 (70.0 -80.0)	0.64
Creatinin (mg/dL)	0.66 (0.58–0.74)	0.69 (0.60–0.79)	0.64 (0.57–0.81)	0.66 (0.58–0.71)	0.55
Uric acid (mg/dL)	4.80 (4.30–5.70)	4.90 (4.40–5.50) ^a^	5.30 (4.40–6.20) ^a^	4.40 (3.35–4.85) ^b^	**4 × 10^−3^**
Total cholesterol (mg/dL)	192 (164–213)	196 (170–228)	183 (163–198)	192 (163–216)	0.19
HDL (mg/dL)	48.0 (39.0–56.0)	50.0 (42.0–58.0)	43.0 (35.0–54.0)	49.0 (45.0–55.0)	0.12
LDL (mg/dL)	117 (91.8–141)	124 (100–151)	106 (85.6–127)	111 (96.1–139)	0.13
Triglycerides (mg/dL)	117 (95.0–177)	115 (83.0–177)	120 (103–205)	103 (85.0–162)	0.33
AST (IU/L)	22.0 (19.0–27.8)	23.5 (20.0–33.8)	20.0 (19.0–24.0)	22.0 (17.5–27.0)	0.25
ALT (IU/L)	21.0 (15.0–28.0)	25.0 (18.0–28.0)	19.0 (15.0–27.0)	18.0 (13.0–31.5)	0.23
GGT (IU/L)	17.5 (12.0–27.0)	21.0 (14.5–37.5)	16.0 (12.0–31.3)	15.0 (10.0–19.5)	0.06
Glucose (mg/dL)	92.0 (88.0–99.0)	93.0 (87.0–100)	92.0 (88.0–101)	91.0 (88.0–96.0)	0.48
Insulin (IU/mL)	67.0 (46.0–96.0)	57.0 (44.0–96.0)	69.0 (44.0–106)	69.0 (48.5–97.0)	0.60
HOMA-IR	1.66 (1.09–2.34)	1.55 (1.00–2.70)	1.72 (1.19–2.71)	1.58 (1.16–2.12)	0.75
Total energy intake (kcal/day)	2085.78 (1583.04–2427.43)	2085.78 (1581.05–2404.53)	2223.51 (1602.68–2579.91)	1772.75 (1575.70–2212.96)	0.39
Total DF (g/1000 kcal/day)	11.5 (9.25–13.9)	11.7 (9.44–13.5)	11.4 (8.40–14.2)	11.4 (9.52–14.5)	0.86
Soluble fiber (g/1000 kcal/day)	3.68 (2.63–4.44)	3.75 (2.66–4.42)	3.66 (2.78–4.83)	3.46 (2.57–4.69)	0.89
Insoluble fiber (g/1000 kcal/day)	6.07 (4.88–7.25)	6.46 (5.31–7.25)	5.81 (4.29–7.90)	6.29 (5.64–7.24)	0.58
Hemicellulose (g/1000 kcal/day)	1.91 (1.61–2.50)	1.92 (1.63–2.34)	2.01 (1.31–2.76)	1.79 (1.79–2.14)	0.48
Cellulose (g/1000 kcal/day)	3.62 (2.24–5.12)	2.94 (2.02–4.69)	3.82 (2.40–6.59)	2.79 (2.21–4.53)	0.22
Lignin (g/1000 kcal/day	0.47 (0.34–0.65)	0.47 (0.34–0.79)	0.40 (0.28–0.63)	0.51 (0.38–0.71)	0.48
AI of dietary fiber; n (%)	25.0 (33.3)	9.0 (33.3)	10.0 (37.0)	6.00 (28.6)	0.83

Values represent median and interquartile range or *n* and percentage. Significant *p*-values are shown in bold. BP: Blood pressure; AST: Aspartate aminotransferase; ALT: Alanine aminotransferase; GGT: Gamma glutamyl transferase; DF: Dietary fiber, AI: Adequate intake. Different superscript letters indicate statistically significant differences between groups after Dunn’s post hoc test.

**Table 3:** Concentration of fecal short-chain fatty acids in adults.

	Total Sample (*n* = 47)	Bacteroides (*n* = 12)	Prevotella (*n* = 26)	Ruminococcaceae (*n* = 9)	*p*
Acetate (mmol/g wet stool)	168 (138–210)	140 (101–186)	181 (153–231)	192 (133–249)	0.10
Propionate (mmol/g wet stool)	133 (107–160)	114 (76–139) ^b^	143 (114–190) ^a^	117 (84.0–144) ^ab^	**0.04**
Butyrate (mmol/g wet stool)	86 (62–119)	67 (39–115)	92.1 (62.8–126)	84.6 (68.3–111)	0.42
Total SCFA (mmol/g wet stool)	389 (310–499)	319 (236–418)	425 (317–509)	350 (307–515)	0.15

Kruskall–Wallis test between enterotypes. Significant *p*-values are shown in bold. Different superscript letters indicate statistically significant differences between groups after a post hoc Dunn’s test. SCFA: Short-chain fatty acids.

## Data Availability

The data that support the findings of this study are available on request from the corresponding author (S.M.-R.).

## Supplementary Materials

The following are available online at https://www.mdpi.com/article/10.3390/nu13113892/s1, Table S1: Habitual nutrient intake in the included school-age children, Table S2: Habitual nutrient intake in the included adults, Table S3: Correlations of dietary fiber intake and metabolic traits in school-age children, Table S4: Correlations of dietary fiber intake and metabolic traits in adults, Table S5: FDR corrected P-values of correlations between dietary fiber intake and metabolic traits in adults, Table S6: FDR corrected P-values of correlations between dietary fiber intake and metabolic traits in children, Table S7: Habitual nutrient intake in children based on Community typing with Dirichlet Multinomial Mixtures, Table S8: Habitual nutrient intake in adults based on Community typing with Dirichlet Multinomial Mixtures, Figure S1: Relative abundance of driver taxa among enterotypes identified by clustering, Figure S2: Alpha diversity estimates within identified enterotypes in adults, Figure S3: Community typing with Dirichlet Multinomial Mixtures in adults, Figure S4: Community typing with Dirichlet Multinomial Mixtures in children.
