# Supplementary material for: Effect of Gut Microbial Enterotypes on the Association between Habitual Dietary Fiber Intake and Insulin Resistance Markers in Mexican Children and Adults
Source: Nutrients. 2021 Oct 29;13(11):3892. doi: 10.3390/nu13113892 (PMC8625328; doi:10.3390/nu13113892)
Supplement: Supplementary file 1 [file nutrients-13-03892-s001.zip › nutrients-1366672-supplementaryy.pdf]

Supplementary data

Gut microbial enterotypes modify the association between habitual dietary fiber intake and insulin resistance markers in Mexican children and adults. Martinez-Medina JN, et al.

Supplementary Table S1. Habitual nutrient intake in the included school-age children.

	**Total Sample (n = 204) Median (IQR)**	**Bacteroides (n = 136) Median (IQR)**	**Prevotella (n = 68) Median (IQR)**	***P***
Total energy intake (kcal/d)	1824.14 (1431.29 - 2201.73)	1846.04 (1440.87 - 2154.09)	1809.95 (1406.36 - 2328.50)	0.85
Fats (%)	38.3 (34.8 - 42.6)	38.2 (35.3 - 43.2)	38.7 (33.8 – 42.0)	0.31
Proteins (%)	14.8 (13.6 - 16.1)	14.8 (13.6 - 16.1)	14.8 (13.7 - 15.9)	0.92
Carbohydrates (%)	46.5 (42.5 - 50.7)	46.6 (41.7 - 50.1)	46.5 (43.0 - 51.2)	0.33
**Fats**				
Dietary cholesterol (mg/1000 kcal/d)	129.4 (108.1 - 152)	130 (111 - 154)	126 (105 - 141)	0.13
Animal fats (g/1000 kcal/d)	25.9 (21.5 - 29.6)	26.1 (22.0 - 29.7)	25.7 (20.7 - 29.3)	0.50
Vegetable fats (g/1000 kcal/d)	16.5 (13.9 - 19.6)	16.4 (13.9 - 20.2)	17.1 (14.3 - 19.1)	0.74
Saturated fats (g/1000 kcal/d)	14.1 (12.6 - 15.5)	14.0 (12.5 - 15.6)	14.2 (12.7 - 15.1)	0.95
Monounsaturated fats (g/1000 kcal/d)	16.1 (14.3 - 17.8)	16.2 (14.5 - 17.8)	16.0 (13.8 - 18.0)	0.69
Polyunsaturated fats (g/1000 kcal/d)	6.96 (5.99 - 8.25)	7.04 (6.04 - 8.31)	6.87 (5.86 - 8.21)	0.50
**Carbohydrates**				
Glucose (g/1000 kcal/d)	7.15 (5.69 - 9.11)	6.96 (5.61 - 9.11)	7.78 (5.85 - 9.17)	0.32
Fructose (g/1000 kcal/d)	10.4 (7.92 - 12.67)	9.78 (7.86 - 12.4)	11.1 (8.18 - 13.6)	0.14
Maltose (g/1000 kcal/d)	0.60 (0.49 - 0.76)	0.59 (0.48 - 0.75)	0.62 (0.50 - 0.79)	0.30
Lactose (g/1000 kcal/d)	9.19 (6.16 - 14.14)	9.09 (6.17 - 13.9)	9.29 (6.16 - 14.7)	0.84
Sucrose (g/1000 kcal/d)	14.6 (11.5 - 17.7)	14.3 (11.2 - 17.7)	15.1 (12.2 - 17.5)	0.71
Starch (g/1000 kcal/d)	27.0 (20.8 - 34.6)	27.0 (20.0 - 35.4)	26.78 (21.0 - 33.8)	0.70

[1] *$P < 0.05$ (U Mann-Whitney test)

Supplementary data

Gut microbial enterotypes modify the association between habitual dietary fiber intake and insulin resistance markers in Mexican children and adults. Martinez-Medina JN, et al.

Supplementary Table S2: Habitual nutrient intake in the included adults.

	**Total sample (n = 75) Median (IQR)**	**Bacteroides (n = 27) Median (IQR)**	**Prevotella (n = 27) Median (IQR)**	**Ruminococcaceae (n = 21) Median (IQR)**	***P***
Total energy intake (kcal/d)	2085.78 (1583.04 - 2427.43)	2085.78 (1581.05- 2404.53)	2223.51 (1602.68 - 2579.91)	1772.75 (1575.70 - 2212.96)	0.39
Fats (%)	33.6 (29.4 – 38.1)	33.6 (29.3 - 39.5)	33.6 (30.3 – 38.1)	33.3 (29.5 – 37.1)	0.76
Proteins (%)	14.3 (13.3 – 16.3)	14.3 (13.7 - 17.3)	14.0 (13.0 - 15.6)	15.5 (12.8 - 16.1)	0.57
Carbohydrates (%)	52.0 (46.9 - 56.0)	52.0 (42.7 - 56.0)	51.9 (46.6 - 58.5)	52.3 (47.7 - 56.2)	0.56
**Fats**					
Dietary cholesterol (mg/1000 kcal/d)	130 (98.5 - 159)	137 (93.3 - 154)	133 (103 - 171)	125 (100 - 157)	0.76
Animal fats (g/1000 kcal/d)	19.1 (15.5 - 23.2)	18.9 (16.4 - 24.0)	19.3 (14.9 - 22.2)	18.3 (15.6 - 22.4)	0.76
Vegetable fats (g/1000 kcal/d)	20.1 (16.8 - 23.1)	20.9 (16.8 - 24.9)	19.5 (14.6 - 22.7)	19.2 (17.6 - 23.5)	0.58
Saturated fats (g/1000 kcal/d)	12.1 (10.3 - 13.4)	12.1 (10.8 - 14.1)	11.9 (10.1 - 13.3)	11.9 (10.0 - 14.1)	0.76
Monounsaturated fats (g/1000 kcal/d)	15.9 (14.0 - 18.0)	15.8 (14.1 - 18.9)	16.3 (13.7 - 20.1)	15.4 (14.4 - 17.3)	0.70
Polyunsaturated fats (g/1000 kcal/d)	7.59 (6.30 - 9.02)	7.45 (6.36 - 10.0)	7.45 (5.73 - 8.13)	8.01 (6.80 - 9.20)	0.54
**Carbohydrates**					
Glucose (g/1000 kcal/d)	7.66 (6.01 - 10.1)	7.91 (6.19 - 11.9)	7.31 (6.01 - 10.5)	7.78 (5.02 - 10.0)	0.91
Fructose (g/1000 kcal/d)	10.1 (8.02 – 14.0)	10.3 (7.94 - 15.6)	9.89 (7.94 – 13.0)	10.2 (8.35 - 13.3)	0.88
Maltose (g/1000 kcal/d)	0.57 (0.46 - 0.73)	0.58 (0.40 - 0.84)	0.55 (0.46 - 0.62)	0.63 (0.48 - 0.76)	0.41
Lactose (g/1000 kcal/d)	3.95 (1.60 - 7.50)	4.62 (1.41 - 8.12)	2.71 (1.53 - 5.95)	4.94 (2.49 - 7.61)	0.35
Sucrose (g/1000 kcal/d)	15.1 (11.3 - 20.6)	15.2 (10.6 - 17.2)	14.5 (11.0 - 24.3)	15.2 (12.3 - 22.8)	0.68
Starch (g/1000 kcal/d)	35.2 (26.9 - 44.2)	34.4 (27.5 - 42.7)	39.0 (29.0 - 47.7)	31.0 (24.7 - 48.8)	0.47

[1] *$P < 0.05$ (Kruskal-wallis test)

Supplementary data

Gut microbial enterotypes modify the association between habitual dietary fiber intake and insulin resistance markers in Mexican children and adults. Martinez-Medina JN, et al.

Supplementary Table S3. Correlations of dietary fiber intake and metabolic traits in school-age children.

	**Total fiber**			**Soluble fiber**			**Insoluble fiber**			**Hemi-cellulose**			**Cellulose**			**Lignin**		
	**rho**	**pAdj**	**p_{FDR}**	**rho**	**pAdj**	**p_{FDR}**	**rho**	**pAdj**	**p_{FDR}**	**rho**	**pAdj**	**p_{FDR}**	**rho**	**pAdj**	**p_{FDR}**	**rho**	**pAdj**	**p_{FDR}**
BMI percentile [a]	-0.05	0.51	0.7	-0.03	0.66	0.75	-0.10	0.17	0.64	-0.04	0.56	0.98	-0.13	0.06	0.32	0.07	0.30	0.85
Waist/hip ratio [b]	0.04	0.55	0.7	0.07	0.36	0.75	-0.11	0.11	0.64	0.01	0.85	0.98	-4x10-	0.95	0.99	0.05	0.46	0.85
Body fat (%) [b]	-0.04	0.57	0.7	-0.03	0.63	0.75	-0.11	0.12	0.64	-0.06	0.39	0.98	-0.10	0.14	0.46	0.06	0.40	0.85
Sistolic BP percentile [c]	-0.08	0.28	0.64	-0.02	0.78	0.8	-0.02	0.83	0.83	-0.05	0.48	0.98	0.02	0.74	0.91	-0.06	0.39	0.85
Diastolic BP percentile [c]	0.02	0.81	0.81	0.03	0.64	0.75	0.03	0.72	0.82	-0.03	0.65	0.98	0.10	0.16	0.46	0.02	0.79	0.89
Uric acid (mg/dL) [d]	-0.04	0.53	0.7	0.06	0.41	0.75	-0.03	0.69	0.82	-0.06	0.41	0.98	0.00	0.99	0.99	-0.02	0.83	0.89
Total cholesterol (mg/dL) [d]	-0.04	0.61	0.7	-0.05	0.47	0.75	-0.04	0.53	0.82	-0.03	0.73	0.98	0.06	0.39	0.69	0.05	0.51	0.85
HDL (mg/dL) [d]	-0.09	0.21	0.64	-0.17	**0.01**	0.16	0.08	0.28	0.64	0.02	0.73	0.98	0.13	0.06	0.32	0.05	0.53	0.85
LDL (mg/dL) [d]	-0.04	0.56	0.7	-0.02	0.80	0.8	-0.05	0.48	0.82	-0.01	0.89	0.98	-0.03	0.66	0.91	0.00	0.99	0.99
Triglycerides (mg/dL) [d]	0.08	0.26	0.64	0.09	0.19	0.75	-0.08	0.25	0.64	-0.02	0.74	0.98	0.16	**0.02**	0.32	-0.03	0.71	0.89
AST (IU/L) [d]	0.10	0.15	0.64	0.07	0.31	0.75	0.04	0.61	0.82	0.00	0.95	0.98	-0.03	0.69	0.91	0.11	0.12	0.52
ALT (IU/L) [d]	0.10	0.16	0.64	0.03	0.64	0.75	0.02	0.78	0.83	0.03	0.63	0.98	-0.07	0.30	0.6	0.17	**0.02**	0.32
GGT (IU/L) [d]	-0.02	0.79	0.81	0.04	0.61	0.75	-0.03	0.65	0.82	0.02	0.73	0.98	-0.10	0.18	0.46	0.02	0.74	0.89
Glucose (mg/dL) [d]	0.05	0.49	0.7	0.05	0.52	0.75	-0.10	0.17	0.64	0.00	0.98	0.98	0.09	0.20	0.46	-0.03	0.68	0.89
Insulin (IU/mL) [d]	-0.08	0.24	0.64	-0.11	0.11	0.69	-0.07	0.33	0.66	-0.01	0.87	0.98	-0.02	0.74	0.91	-0.11	0.13	0.52
HOMA-IR [d]	-0.08	0.27	0.64	-0.11	0.13	0.69	-0.08	0.28	0.64	-0.01	0.86	0.98	-0.02	0.82	0.94	-0.11	0.13	0.52

[1]Spearman test. Statistical significance pAdj < 0.05

[2]a without adjustment.

[3]b adjusted by age and sex.

[4]c adjusted by body fat (%).

[5]d adjusted by age, sex, and body fat (%).

[6]Fiber data presented as g/1000 kcal/d; n = 204

[7]BP: Blood pressure; AST: Aspartate aminotransferase; ALT: Alanine aminotransferase; GGT: Gamma glutamyl transferase

Supplementary data

Gut microbial enterotypes modify the association between habitual dietary fiber intake and insulin resistance markers in Mexican children and adults. Martinez-Medina JN, et al.

Supplementary Table S4. Correlations of dietary fiber intake and metabolic traits in adults.

	**Total fiber**			**Soluble fiber**			**Insoluble fiber**			**Hemi-cellulose**			**Cellulose**			**Lignin**		
	**rho**	**pAdj**	**p_{FDR}**	**rho**	**pAdj**	**p_{FDR}**	**rho**	**pAdj**	**p_{FDR}**	**rho**	**pAdj**	**p_{FDR}**	**rho**	**pAdj**	**p_{FDR}**	**rho**	**pAdj**	**p_{FDR}**
BMI [a]	-0.02	0.90	0.96	-0.04	0.74	0.85	0.05	0.65	0.96	0.07	0.54	0.66	0.02	0.90	0.96	-0.06	0.64	0.93
Waist/hip ratio [a]	-0.20	0.10	0.73	-0.13	0.29	0.85	-0.25	**0.03**	0.43	-0.22	0.06	0.48	0.00	0.97	0.97	-0.31	**0.01**	0.16
Body fat (%) [a]	0.13	0.30	0.73	0.05	0.67	0.85	0.17	0.15	0.54	0.28	**0.02**	0.32	0.12	0.32	0.76	0.03	0.79	0.96
Sistolic BP (mmHg) [b]	-0.08	0.52	0.84	-0.07	0.55	0.85	-0.02	0.89	0.96	-0.06	0.60	0.69	0.03	0.79	0.95	0.04	0.73	0.96
Diastolic BP (mmHg) [b]	-0.15	0.22	0.73	-0.18	0.14	0.85	-0.01	0.92	0.96	-0.05	0.71	0.71	-0.11	0.38	0.76	0.01	0.96	0.96
Uric acid (mg/dL) [b]	0.06	0.62	0.84	0.15	0.23	0.85	0.01	0.94	0.96	0.09	0.48	0.66	0.10	0.42	0.76	0.02	0.84	0.96
Total cholesterol (mg/dL) [b]	-0.18	0.14	0.73	-0.13	0.28	0.85	-0.01	0.94	0.96	-0.10	0.40	0.66	-0.11	0.37	0.76	-0.15	0.23	0.46
HDL (mg/dL) [b]	0.05	0.71	0.86	-0.06	0.62	0.85	0.21	0.08	0.43	0.15	0.22	0.64	0.03	0.83	0.95	0.17	0.16	0.45
LDL (mg/dL) [b]	-0.19	0.11	0.73	-0.12	0.34	0.85	-0.08	0.51	0.89	-0.09	0.45	0.66	-0.06	0.60	0.89	-0.18	0.13	0.45
Triglycerides (mg/dL) [b]	-0.08	0.54	0.84	-0.01	0.91	0.95	0.01	0.96	0.96	-0.17	0.15	0.64	-0.10	0.43	0.76	-0.14	0.26	0.46
AST (IU/L) [b]	-0.02	0.85	0.96	-0.01	0.95	0.95	-0.04	0.76	0.96	-0.08	0.50	0.66	0.04	0.73	0.95	0.02	0.91	0.96
ALT (IU/L) [b]	-0.06	0.63	0.84	-0.06	0.63	0.85	-0.09	0.45	0.89	-0.05	0.67	0.71	-0.19	0.12	0.76	-0.06	0.61	0.93
GGT (IU/L) [b]	-0.13	0.31	0.73	-0.10	0.41	0.85	-0.13	0.29	0.77	-0.15	0.22	0.64	-0.16	0.19	0.76	-0.21	0.09	0.45
Glucose (mg/dL) [b]	0.00	0.98	0.98	0.04	0.74	0.85	-0.11	0.38	0.87	-0.12	0.32	0.64	0.14	0.26	0.76	-0.22	0.07	0.45
Insulin (IU/mL) [b]	-0.10	0.41	0.82	-0.06	0.65	0.85	-0.21	0.08	0.43	-0.13	0.29	0.64	-0.06	0.61	0.89	-0.14	0.26	0.46
HOMA-IR [b]	-0.12	0.32	0.73	-0.10	0.41	0.85	-0.17	0.17	0.54	-0.14	0.24	0.64	-0.11	0.37	0.76	-0.17	0.17	0.45

[1]Spearman test. Statistical significance pAdj < 0.05

[2]a adjusted by age and sex

[3]b adjusted by age, sex and body fat (%).

[4]Fiber data presented as g/1000 kcal/d; n = 75

[5]BP: Blood pressure; AST: Aspartate aminotransferase; ALT: Alanine aminotransferase; GGT: Gamma glutamyl transferase

Supplementary data

Gut microbial enterotypes modify the association between habitual dietary fiber intake and insulin resistance markers in Mexican children and adults. Martinez-Medina JN, et al.

Supplementary Table S5. FDR corrected P-values of correlations between dietary fiber intake and metabolic traits in children

	Bacteroides						Prevotella					
	**Total fiber**	**Soluble fiber**	**Insoluble fiber**	**Hemicellulose**	**Cellulose**	**Lignin**	**Total fiber**	**Soluble Fiber**	**Insoluble fiber**	**Hemicellulose**	**Cellulose**	**Lignin**
BMI	0.73	0.94	0.93	0.79	0.60	1.00	0.89	0.82	0.42	0.90	0.94	0.62
Waist/hip ratio	0.73	0.77	0.93	0.95	0.86	1.00	0.89	0.95	0.42	0.90	0.72	0.62
Body fat (%)	0.84	0.94	0.93	0.79	0.60	1.00	0.89	0.82	0.42	0.90	0.94	0.73
Sistolic BP (mmHg)	0.84	0.85	0.93	0.79	0.86	1.00	0.89	0.57	0.43	0.90	0.72	0.43
Diastolic BP (mmHg)	0.84	0.85	0.93	0.79	0.86	1.00	0.89	0.82	0.42	0.88	0.26	0.96
Uric acid (mg/dL)	0.73	0.94	0.93	0.79	0.86	1.00	0.89	0.77	0.87	0.90	0.96	0.62
Total cholesterol (mg/dL)	0.93	0.98	0.93	0.88	0.86	1.00	0.89	0.66	0.42	0.90	0.73	0.62
HDL (mg/dL)	0.73	0.43	0.93	0.95	0.86	1.00	0.89	0.57	0.79	0.90	0.26	0.73
LDL (mg/dL)	0.84	0.98	0.99	0.79	0.86	1.00	0.89	0.77	0.42	0.90	0.72	0.62
Triglycerides (mg/dL)	0.73	0.77	0.99	0.79	0.60	1.00	0.89	0.57	0.42	0.88	0.72	0.62
TG_HDL_ratio	0.73	0.43	0.93	0.79	0.86	1.00	0.89	0.57	0.42	0.88	0.72	0.62
AST (IU/L)	0.84	0.85	0.93	0.79	0.86	1.00	0.79	0.82	0.42	0.90	0.96	0.26
ALT (IU/L)	0.84	0.98	0.93	0.95	0.86	1.00	0.79	0.77	0.43	0.90	0.72	0.00
GGT (IU/L)	0.73	0.98	0.93	0.79	0.86	1.00	0.89	0.66	0.42	0.62	0.72	0.43
Glucose (mg/dL)	0.84	0.94	0.93	0.96	0.60	1.00	0.89	0.82	0.42	0.90	0.96	0.62
Insulin (IU/mL)	0.84	0.85	0.99	0.79	0.86	1.00	0.79	0.57	0.42	0.17	0.96	0.43
HOMA-IR	0.84	0.85	0.99	0.79	0.86	1.00	0.85	0.57	0.42	0.17	0.96	0.43

Supplementary data

Gut microbial enterotypes modify the association between habitual dietary fiber intake and insulin resistance markers in Mexican children and adults. Martinez-Medina JN, et al.

Supplementary Table S6. FDR corrected P-values of correlations between dietary fiber intake and metabolic traits in adults enterotypes

	Bacteroides						Prevotella						Ruminococcaceae					
	**Total fiber**	**Soluble fiber**	**Insoluble fiber**	**Hemi-cellulose**	**Cellulose**	**Lignin**	**Total fiber**	**Soluble fiber**	**Insoluble fiber**	**Hemi-cellulose**	**Cellulose**	**Lignin**	**Total fiber**	**Soluble fiber**	**Insoluble fiber**	**Hemi-cellulose**	**Cellulose**	**Lignin**
BMI	0.85	0.93	0.90	0.93	0.17	0.93	0.96	0.95	0.77	0.84	0.14	0.86	0.66	0.60	1.00	0.98	0.71	0.77
Waist/hip ratio	0.57	0.93	0.57	0.75	1.00	0.17	0.96	0.91	0.59	0.41	0.43	0.57	0.53	0.53	1.00	0.98	0.60	0.77
Body fat (%)	0.82	0.93	0.57	0.93	0.40	0.93	0.26	0.51	0.29	0.14	0.96	0.24	0.53	0.34	1.00	0.68	0.71	0.34
Sistolic BP (mmHg)	0.83	0.98	0.60	0.83	0.94	0.93	0.80	0.70	0.77	0.75	0.96	0.68	0.66	0.60	1.00	0.98	0.43	0.94
Diastolic BP (mmHg)	0.82	0.93	0.57	0.75	0.94	1.00	0.62	0.70	0.77	0.84	0.86	0.68	0.53	0.34	1.00	0.98	0.17	0.77
Uric acid (mg/dL)	0.82	0.93	0.57	0.93	0.95	0.93	0.96	0.96	0.77	0.84	0.49	0.68	0.53	0.34	1.00	0.98	0.40	0.60
Total cholesterol (mg/dL)	0.43	0.93	0.57	0.26	0.40	0.85	0.80	0.62	0.77	0.83	0.96	0.84	0.66	0.78	1.00	0.98	0.71	0.88
HDL (mg/dL)	0.82	0.98	0.57	0.75	0.72	0.93	0.77	0.95	0.29	0.14	0.27	0.09	0.66	0.78	1.00	0.85	0.71	0.77
LDL (mg/dL)	0.17	0.93	0.34	0.26	0.40	0.34	1.00	0.95	0.77	0.83	0.78	0.84	0.66	0.82	1.00	0.98	0.71	0.77
Triglycerides (mg/dL)	0.83	0.93	0.83	0.75	0.95	0.93	0.62	0.70	0.59	0.36	0.14	0.24	0.53	0.53	0.34	0.98	0.71	0.77
TG_HDL_ratio	0.82	0.93	0.57	0.75	0.94	0.93	0.62	0.85	0.29	0.14	0.14	0.09	0.53	0.53	0.34	0.98	0.71	0.77
AST (IU/L)	0.82	0.97	0.57	0.93	0.72	0.93	0.17	0.34	0.29	0.32	0.43	0.57	0.66	0.78	1.00	0.98	0.95	0.99
ALT (IU/L)	0.82	0.93	0.57	0.93	0.66	1.00	0.17	0.51	0.29	0.14	0.14	0.20	0.66	0.79	1.00	0.98	0.60	0.88
GGT (IU/L)	0.82	0.93	0.61	0.51	0.94	0.93	0.82	0.95	0.77	0.36	0.68	0.28	0.64	0.53	1.00	0.98	0.00	0.77
Glucose (mg/dL)	0.83	0.99	0.57	0.75	0.94	0.93	0.96	0.95	0.77	0.68	0.96	0.51	0.53	0.53	1.00	0.98	0.71	0.88
Insulin (IU/mL)	0.83	0.93	0.90	0.75	0.40	1.00	0.26	0.51	0.29	0.14	0.14	0.09	0.66	0.62	1.00	0.98	0.95	0.77
HOMA-IR	0.83	0.93	0.83	0.93	0.40	1.00	0.31	0.51	0.29	0.14	0.14	0.09	0.66	0.80	1.00	0.98	0.71	0.77

Supplementary data

Gut microbial enterotypes modify the association between habitual dietary fiber intake and insulin resistance markers in Mexican children and adults. Martinez-Medina JN, et al.

Supplementary Table S7: Habitual nutrient intake in children based on Community typing with Dirichlet Multinomial Mixtures.

	**Total sample (n = 204) Median (IQR)**	**Bacteroides (n = 83) Median (IQR)**	**Prevotella (n = 58) Median (IQR)**	**Bacteroides 2 (n = 63) Median (IQR)**	***P**
Total energy intake (kcal/d)	1824.14 (1431.29 - 2201.73)	1742.60 (1402.2-2124.65)	1856.10 (1419.94-2386.05)	1864.23 (1449.09-2167.44)	0.63
Fats (%)	38.3 (34.8 - 42.6)	38.3 (35.3-43.7)	38.4 (34.0-42.0)	38.4 (35.0-41.8)	0.54
Proteins (%)	14.8 (13.6 - 16.1)	14.9 (13.6-16.1)	14.7 (13.7-15.9)	14.6 (13.5-16.0)	0.77
Carbohydrates (%)	46.5 (42.5 - 50.7)	46.6 (41.2-50.1)	46.9 (42.8-51.1)	46.4 (43.3-51.1)	0.55
**Fats**					
Dietary cholesterol (mg/1000 kcal/d)	129.4 (108.1 - 152)	131.1 (110.9-162.9)	129.7 (109.5-143.4)	127.7 (102.7-150.5)	0.42
Animal fats (g/1000 kcal/d)	25.9 (21.5 - 29.6)	26.2 (22.4-30.1)	25.8 (21.1-29.6)	25.4 (20.8-29.2)	0.24
Vegetable fats (g/1000 kcal/d)	16.5 (13.9 - 19.6)	16.1 (13.9-20.2)	16.5 (13.7-19.1)	17.1 (14.0-19.6)	0.74
Saturated fats (g/1000 kcal/d)	14.1 (12.6 - 15.5)	14.5 (12.4-16.0)	14.2 (12.9-15.7)	13.5 (12.3-14.8)	0.54
Monounsaturated fats (g/1000 kcal/d)	16.1 (14.3 - 17.8)	7.12 (6.09-8.42)	7.01 (5.89-8.24)	6.86 (5.98-8.00)	0.65
Polyunsaturated fats (g/1000 kcal/d)	6.96 (5.99 - 8.25)	16.4 (14.5-18.1)	16.0 (13.8-17.9)	15.9 (14.0-17.4)	0.90
**Carbohydrates**					
Glucose (g/1000 kcal/d)	7.15 (5.69 - 9.11)	6.84 (5.17-9.34)	7.27 (5.72-8.82)	7.44 (5.97-9.44)	0.36
Fructose (g/1000 kcal/d)	10.4 (7.92 - 12.67)	9.62 (7.18-12.3)	10.6 (7.98-12.3)	10.9 (8.25-13.6)	0.68
Maltose (g/1000 kcal/d)	0.60 (0.49 - 0.76)	0.60 (0.49-0.75)	0.60 (0.49-0.75)	0.60 (0.49-0.81)	0.80
Lactose (g/1000 kcal/d)	9.19 (6.16 - 14.14)	9.28 (6.12-14.5)	9.80 (6.35-15.79)	8.47 (6.04-13.4)	0.08
Sucrose (g/1000 kcal/d)	14.6 (11.5 - 17.7)	13.9 (11.2-17.7)	15.1 (11.8-17.2)	15.1 (11.7-18.0)	0.58
Starch (g/1000 kcal/d)	27.0 (20.8 - 34.6)	26.0 (19.4-35.7)	27.9 (21.1-33.9)	27.3 (21.9-35.2)	0.58
**Fiber**					
Total DF (g/1000kcal/day)	10.4 (8.73 – 11.8)	10.0 (8.66-11.3)	10.1 (8.29-11.9)	10.8 (9.14-12.0)	0.16
Soluble fiber (g/1000kcal/day)	2.95 (2.44 – 3.53)	2.93 (2.44-3.35)	2.84 (2.34-3.55)	3.10 (2.65-3.73)	0.17
Insoluble fiber (g/1000kcal/day)	5.67 (4.94 – 6.59)	5.67 (4.76-6.46)[a]	5.33 (4.71-6.45)[a]	5.83 (5.39-7.29)[b]	**0.01***
Hemicellulose (g/1000kcal/day)	1.82 (1.53 - 2.31)	1.76 (1.48-2.19)	1.80 (1.54-2.15)	1.95 (1.58-2.61)	0.10
Cellulose (g/1000kcal/day)	2.67 (1.96 - 4.24)	2.54 (2.01-4.05)	2.82 (1.69-4.28)	2.74 (2.04-4.45)	0.54
Lignin (g/1000kcal/day)	0.46 (0.37 - 0.57)	0.46 (0.35-0.58)	0.44 (0.35-0.57)	0.48 (0.40-0.58)	0.56
AI of dietary fiber; n (%)	23.0 (11.2)	5 (21.7)	8 (34.8)	10 (43.5)	0.14

[1] *$P < 0.05$ (Kruskal-wallis test). [abc] Different letters indicate statistically significant differences between groups after a post hoc Dunn's test.

Supplementary data

Gut microbial enterotypes modify the association between habitual dietary fiber intake and insulin resistance markers in Mexican children and adults. Martinez-Medina JN, et al.

Supplementary Table S8: Habitual nutrient intake in adults based on Community typing with Dirichlet Multinomial Mixtures.

	Total sample (n = 75) Median (IQR)	Bacteroides (n = 27) Median (IQR)	Prevotella (n = 23) Median (IQR)	Bacteroides 2 (n = 25) Median (IQR)	*P
Total energy intake (kcal/d)	2085.78 (1583.04 - 2427.43)	1777.3 (1568.36-2199.12)	2327.71 (1921.90-2582.13)	2085.78 (1651.36-2445.29)	0.25
Fats (%)	33.6 (29.4 – 38.1)	33.3 (31.3-37.3)	33.6 (28.9-38.1)	33.6 (29.2-39.3)	0.90
Proteins (%)	14.3 (13.3 – 16.3)	15.5 (13.1-16.3)	14.0 (13.0-14.0)	14.3 (13.5-17.5)	0.59
Carbohydrates (%)	52.0 (46.9 - 56.0)	51.9 (47.3-54.8)	51.6 (46.6-58.7)	52.1 (44.5-56.6)	0.76
**Fats**					
Dietary cholesterol (mg/1000 kcal/d)	130 (98.5 - 159)	130.7 (98.5-159.8)	133.6 (107.0-189.0)	129.0 (88.5-151.0)	0.50
Animal fats (g/1000 kcal/d)	19.1 (15.5 - 23.2)	19.1 (16.0-22.1)	20.0 (14.9-23.9)	18.7 (16.3-23.7)	0.91
Vegetable fats (g/1000 kcal/d)	20.1 (16.8 - 23.1)	18.4 (16.9-22.8)	20.1 (14.6-23.3)	20.5 (16.1-24.0)	0.79
Saturated fats (g/1000 kcal/d)	12.1 (10.3 - 13.4)	12.1 (10.0-14.2)	11.9 (10.1-13.0)	12.1 (10.9-13.7)	0.74
Monounsaturated fats (g/1000 kcal/d)	15.9 (14.0 - 18.0)	15.9 (14.7-17.8)	16.5 (13.6-20.1)	15.6 (13.9-18.1)	0.89
Polyunsaturated fats (g/1000 kcal/d)	7.59 (6.30 - 9.02)	7.67 (6.62-9.08)	7.59 (5.73-8.94)	7.45 (6.34-9.51)	0.80
**Carbohydrates**					
Glucose (g/1000 kcal/d)	7.66 (6.01 - 10.1)	7.78 (5.05-9.88)	7.04 (6.01-10.5)	7.91 (6.08-12.8)	0.93
Fructose (g/1000 kcal/d)	10.1 (8.02 – 14.0)	10.2 (8.31-12.6)	9.89 (7.94-12.9)	10.3 (7.89-15.7)	0.92
Maltose (g/1000 kcal/d)	0.57 (0.46 - 0.73)	0.55 (0.48-0.74)	0.55 (0.46-0.62)	0.60 (0.40-0.84)	0.39
Lactose (g/1000 kcal/d)	3.95 (1.60 - 7.50)	3.81 (1.83-7.54)	2.71(1.52-5.95)	5.35 (1.75-8.42)	0.43
Sucrose (g/1000 kcal/d)	15.1 (11.3 - 20.6)	14.9 (10.6-22.2)	15.2 (11.3-24.3)	15.2 (10.9-17.1)	0.77
Starch (g/1000 kcal/d)	35.2 (26.9 - 44.2)	31.3. (23.9-46.7)	39.0 (30.4-47.7)	34.4 (27.2-42.9)	0.26
**Fiber**					
Total DF (g/1000kcal/day)	11.5 (9.25 - 13.9)	10.81 (9.16-13.37)	11.9 (8.64-14.58)	11.65 (9.61-13.77)	0.83
Soluble fiber (g/1000kcal/day)	3.68 (2.63 - 4.44)	3.10 (2.44-4.37)	3.67 (2.97-5.16)	3.75 (2.84-4.40)	0.45
Insoluble fiber (g/1000kcal/day)	6.07 (4.88 - 7.25)	6.05 (5.47-7.21)	5.82 (4.35-8.57)	6.26 (5.20-7.23)	0.95
Hemicellulose (g/1000kcal/day)	1.91 (1.61 - 2.50)	1.75 (1.55-2.19)	2.03 (1.31-2.99)	1.92 (1.67-2.37)	0.35
Cellulose (g/1000kcal/day)	3.62 (2.24 - 5.12)	3.09 (2.22-4.89)	3.74 (2.32-5.79)	3.33 (2.14-4.77)	0.68
Lignin (g/1000kcal/day)	0.47 (0.34 - 0.65)	0.47 (0.37-0.66)	0.40 (0.34-0.73)	0.47 (0.34-0.71)	0.88
AI of dietary fiber; n (%)	25.0 (33.3)	7 (28.0)	9 (36.0)	9 (36.0)	0.58

[1] *$P < 0.05$ (Kruskal-wallis test. [abc] Different letters indicate statistically significant differences between groups after a post hoc Dunn's test.

Supplementary data

Gut microbial enterotypes modify the association between habitual dietary fiber intake and insulin resistance markers in Mexican children and adults. Martinez-Medina JN, et al.

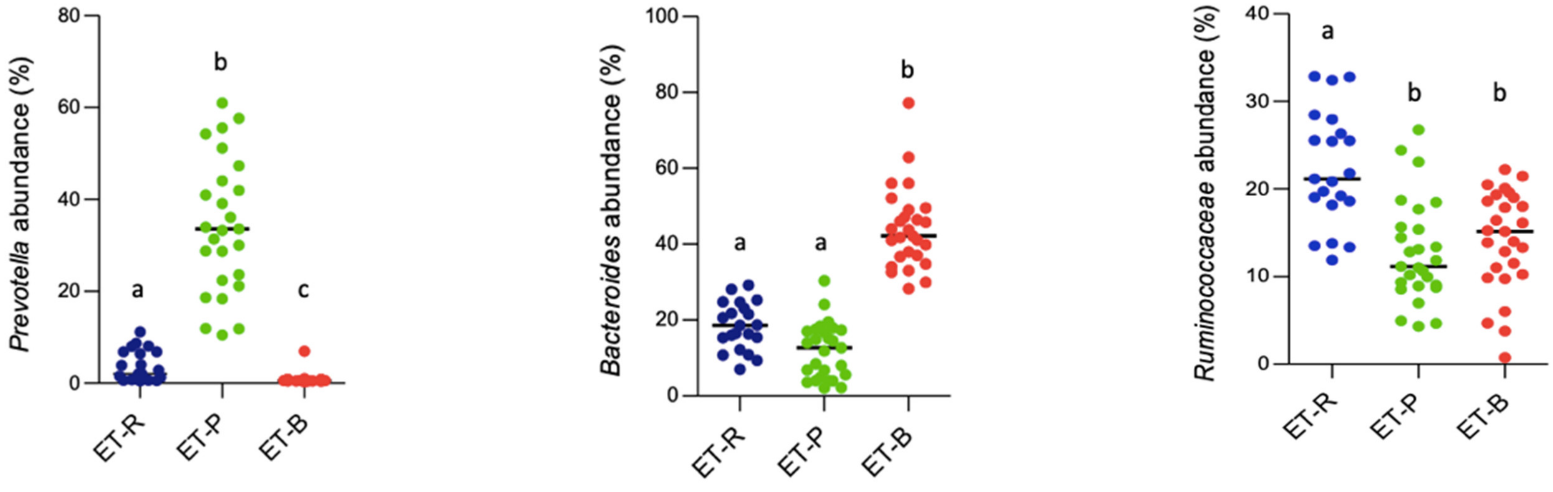

a) b) c)

Supplementary Figure S1. Relative abundance of driver taxa among enterotypes (**a**) *Prevotella,* (**b**) *Bacteroides*, and (**c**) *Ruminococcaceae.* ET-R: *Ruminococacceae* enterotype; ET-P: *Prevotella* enterotype, ET-B: *Bacteroides* enterotype. Data are presented as median and IQR; ET-R: n = 21; ET-P: n = 27 and ET-B: n = 27. [abc] Different letters indicate statistically significant differences between groups after a post hoc Dunn's test.

__Supplementary data__

Gut microbial enterotypes modify the association between habitual dietary fiber intake and insulin resistance markers in Mexican children and adults. Martinez-Medina JN, et al.

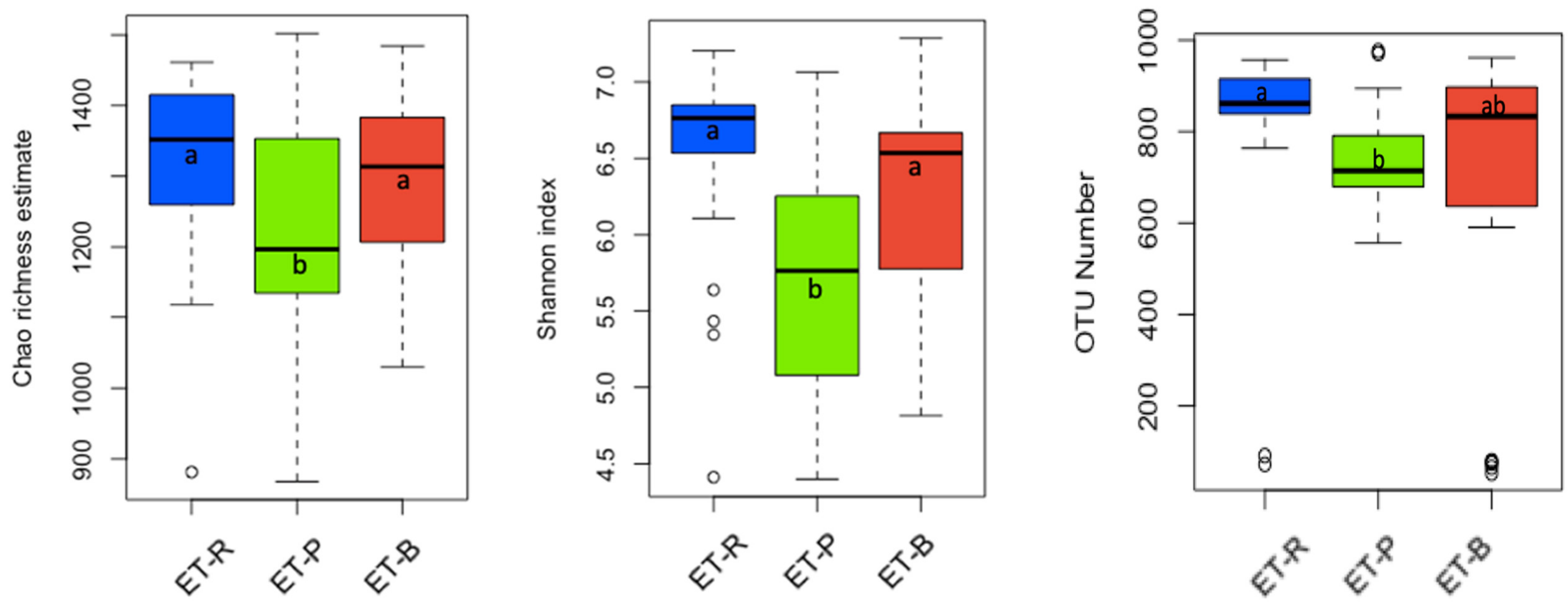

a) **b)** **c)**

Supplementary Figure S2. Alpha diversity estimates within identified enterotypes in adults. **(a)** Chao richness estimate, **(b)** Shannon index, **(c)** Observed OTUs. ET-R: *Ruminococacceae* enterotype; ET-P: *Prevotella* enterotype, ET-B: *Bacteroides* enterotype. Data are presented as median and IQR; ET-R: n = 21; ET-P: n = 27 and ET-B: n = 27. [ab] Different letters indicate statistically significant differences between groups after a post hoc Dunn's test.

<u>Supplementary data</u>

Gut microbial enterotypes modify the association between habitual dietary fiber intake and insulin resistance markers in Mexican children and adults. Martinez-Medina JN, et al.

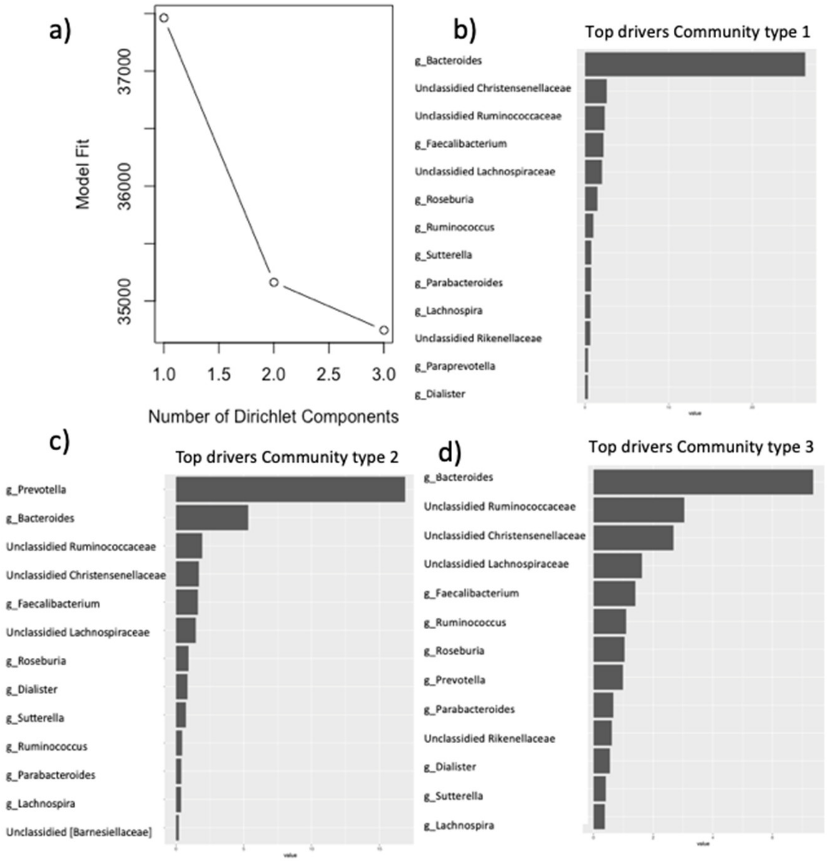

Supplementary Figure S3. Community typing with Dirichlet Multinomial Mixtures in children. **(a)** Number of Optimal Dirichlet Components, **(b)** Top driver taxa: community type 1, **(c)** Top driver taxa: community type 2, **(d)** Top driver taxa: community type 3.

Supplementary data

Gut microbial enterotypes modify the association between habitual dietary fiber intake and insulin resistance markers in Mexican children and adults. Martinez-Medina JN, et al.

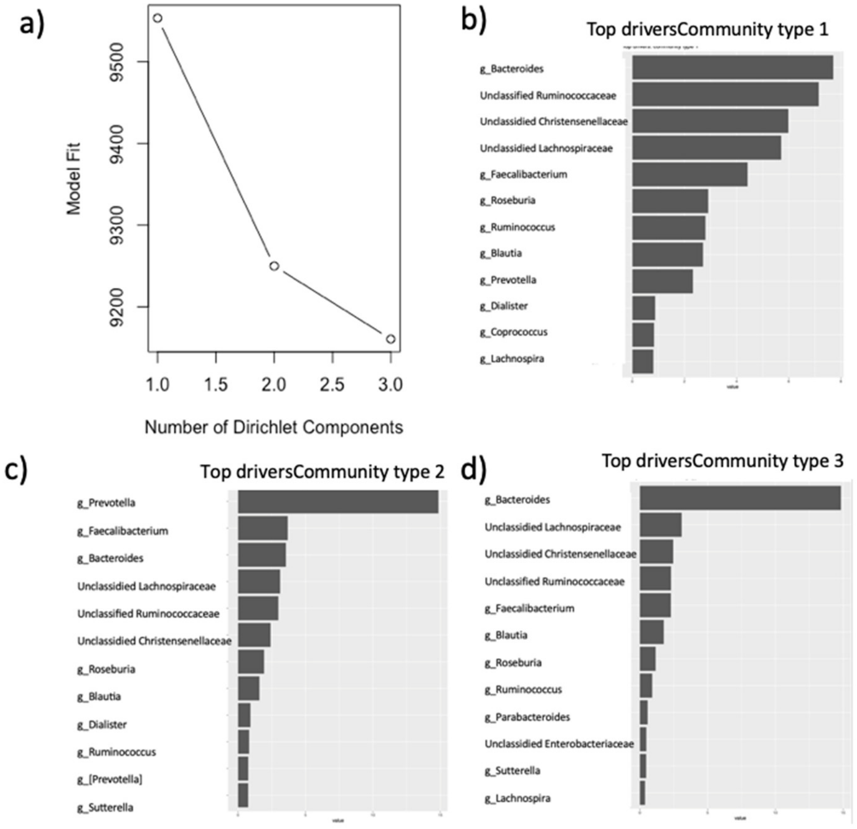

Supplementary Figure S4. Community typing with Dirichlet Multinomial Mixtures in adults. **(a)** Number of Optimal Dirichlet Components, **(b)** Top driver taxa: community type 1, **(c)** Top driver taxa: community type 2, **(d)** Top driver taxa: community type 3.
